# Effects of esketamine on postoperative rebound pain in patients undergoing unilateral total knee arthroplasty: a single-center, randomized, double-blind, placebo-controlled trial protocol

**DOI:** 10.3389/fneur.2023.1179673

**Published:** 2023-04-27

**Authors:** Youzhuang Zhu, Qun Li, Guilin Liu, Fang Sheng, Xiaotian Zhang, Lili Jiang, Shaona Li, Jianshuai He, Zhijin Zou, Baobo Zhang, Changyao Wang, Xin Jiang, Yang Zhao

**Affiliations:** ^1^Department of Anesthesiology, The Affiliated Hospital of Qingdao University, Qingdao, China; ^2^Department of Joint Surgery, The Affiliated Hospital of Qingdao University, Qingdao, China; ^3^Phase I Clinical Trial Center, The Affiliated Hospital of Qingdao University, Qingdao, China

**Keywords:** total knee arthroplasty, esketamine, rebound pain, postoperative acute pain, hyperalgesia

## Abstract

**Introduction:**

Rebound pain, transient and acute postoperative pain after the disappearance of regional block anesthesia, has been a concern in recent years. Insufficient preemptive analgesia and hyperalgesia induced by regional block are the main mechanisms. At present, the evidence for the treatment of rebound pain is limited. The esketamine, as an antagonist of the N-methyl-D-aspartate receptor, has been proven to prevent hyperalgesia. Therefore, this trial aims to evaluate the impact of esketamine on postoperative rebound pain in patients undergoing total knee arthroplasty.

**Methods/design:**

This study is a single-center, prospective, double-blind, randomized, placebo-controlled trial. Participants who plan to undergo total knee arthroplasty will be randomly assigned to the esketamine group (*N* = 178) and placebo group (*N* = 178) in a ratio of 1:1. This trial aims to evaluate the impact of esketamine on postoperative rebound pain in patients undergoing total knee arthroplasty. The primary outcome of this trial is the incidence of rebound pain within 12 h after the operation in the esketamine group and the placebo group. The secondary outcome will be to compare (1) the incidence of rebound pain 24 h after the operation; (2) the time to enter the pain cycle for the first time within 24 h after the procedure; (3) the first time of rebound pain occurred within 24 h after surgery; (4) the modified rebound pain score; (5) NRS score under rest and exercise at different time points; (6) the cumulative opioid consumption at different time points; (7) patient’s prognosis and knee joint function evaluation; (8) blood glucose and cortisol concentration; (9) patient’s satisfaction score; (10) adverse reactions and adverse events.

**Discussion:**

The effect of ketamine on preventing postoperative rebound pain is contradictory and uncertain. The affinity of esketamine to the N-methyl-D-aspartate receptor is about four times higher than levo-ketamine, the analgesic effect is 3 times higher than levo-ketamine, and there are fewer adverse mental reactions. To our knowledge, there is no randomized controlled trial to verify the impact of esketamine on postoperative rebound pain in patients undergoing total knee arthroplasty. Therefore, this trial is expected to fill an important gap in relevant fields and provide novel evidence for individualized pain management.

**Clinical Trial Registration:**

http://www.chictr.org.cn, identifier ChiCTR2300069044.

## 1. Introduction

The term ‘rebound pain’ was recently introduced to describe the temporary, acute postoperative pain that occurs after regional anesthesia sensory block has worn off, and which can be observed following peripheral nerve block and intraspinal anesthesia (1). To date, there is no clear definition of rebound pain. To better quantify rebound pain, Barry et al. (2) proposed a standardized reporting method: if the pain level transitions from well-controlled [numerical rating scale (NRS) ≤ 3] to severe (NRS ≥ 7) within 24 h after regional block, rebound pain is considered to have occurred. The mechanism of rebound pain is unclear, but may be explained by relatively sudden nociceptive pain from insufficient preemptive analgesia or regional block-induced hyperalgesia (1, 3). Other factors may include local anesthetic neurotoxicity, withdrawal reactions, potential pain-promoting effects, and individual or surgical factors (4).

Total knee arthroplasty (TKA) uses artificial materials to replace the knee joint to alleviate and eliminate inflammatory disease, pain, and tissue deformity. However, TKA also causes significant trauma and severe bone, soft tissue, and nerve damage. After TKA, 60% of patients experienced severe knee pain, and 30% experienced moderate pain (5). In China, the Consensus of Experts on Postoperative Pain Management for Adults has suggested that multimodal analgesia should be adopted for TKA. Multimodal analgesia includes non-drug and drug measures. The latter combines several drugs and drug delivery routes, including preventive analgesia, peripheral nerve block, patient-controlled analgesia, periarticular mixed drug injection analgesia, and oral/intravenous use of non-steroidal anti-inflammatory drugs and opioids.

In our medical center, single subarachnoid block anesthesia, instead of general anesthesia, has become the standard of care for TKA. It is relatively low-cost and simple to administer, has good analgesic effects, has a high safety rating, and has minimal cardiopulmonary effects. It is worth noting that subarachnoid block anesthesia, peripheral nerve block, and mixed drug injection around the joint are all regional block types, which can lead to transient, acute postoperative pain after wearing off (i.e., rebound pain). As an acute postoperative pain, rebound pain may cause adverse effects. It often occurs at night, eroding patient sleep quality and seriously affecting rehabilitation (6). Patients who are unaware of this adverse effect and experience unexpected pain may increase their use of oral medications, leading to further opioid consumption and increased emergency room visits (6). Rebound pain also reduces the benefits of a regional block, lowers patient satisfaction, and increases postoperative care costs (6).

Evidence on rebound pain treatment is limited. In patients undergoing arthroscopic rotator cuff repair, dexamethasone added around the nerve with ropivacaine to block the scalene muscle space can reduce pain aggravation after subsidence of the regional block and reduce rebound pain incidence in the first postoperative week (7). Dexamethasone may make the nerve block fade more smoothly, extend its duration, and have anti-inflammatory effects. However, using dexamethasone as an auxiliary anesthetic for a peripheral nerve block is an over-indication, the long-term safety of which has not been evaluated. High-dose peripheral dexamethasone may also lead to a risk of wound infection and neurotoxicity (8). Perineural application of dexmedetomidine in arthroscopic rotator cuff repair can reduce postoperative pain scores and delay rebound pain during the first 48 post-surgical hours (9, 10). Combining two or more nerve blocks and prolonging the block duration significantly reduces rebound pain and oral opioid use (11–13). However, combined use of nerve blocks may increase the risk of local anesthetic poisoning and nerve injury. Thus, continuous peripheral nerve block is more suitable for inpatients and challenging for use in those who are discharged, for whom it means considerable workload and increased economic burden (8).

N-methyl-D-aspartate (NMDA), an excitatory glutamate receptor in the neuronal membrane, plays a crucial role in developing anti-central sensitization. Ketamine is a non-competitive NMDA antagonist with a 2–4-h half-life. It acts on brain and spinal cord neurons and has been used to reduce opioid-induced hyperalgesia. The affinity of esketamine to NMDA receptors is about four times higher, and its analgesic effects three times higher, compared with levo-ketamine, and it has fewer adverse mental effects. Borneman-Cimenti et al. (14) showed that esketamine reduces opioid consumption post-surgery, reduces the pain sensitization area, and improves rehabilitation quality. Touil et al. (15) asserted that ketamine did not prevent severe rebound pain after peripheral nerve block, and that the mechanism of rebound pain might not involve central sensitization (15). The few studies assessing rebound pain treatment with ketamine or esketamine have been contradictory or unclear. Therefore, exploring whether anti-hyperalgesia esketamine can reduce rebound pain occurrence after regional block is highly significant. This prospective, randomized controlled study is expected to fill an important gap in relevant fields and provide novel evidence for individualized pain management.

Objectives: The study described herein will be a single-center, prospective, double-blind, randomized controlled trial, with an aim to explore the effects of esketamine on postoperative rebound pain in patients undergoing TKA. Our hypothesis is that compared with the placebo group, continuous infusion of esketamine during surgery reduces the incidence of rebound pain during the first 12 h after TKA surgery.

## 2. Method

### 2.1. Design and setting

**Figure 1:**
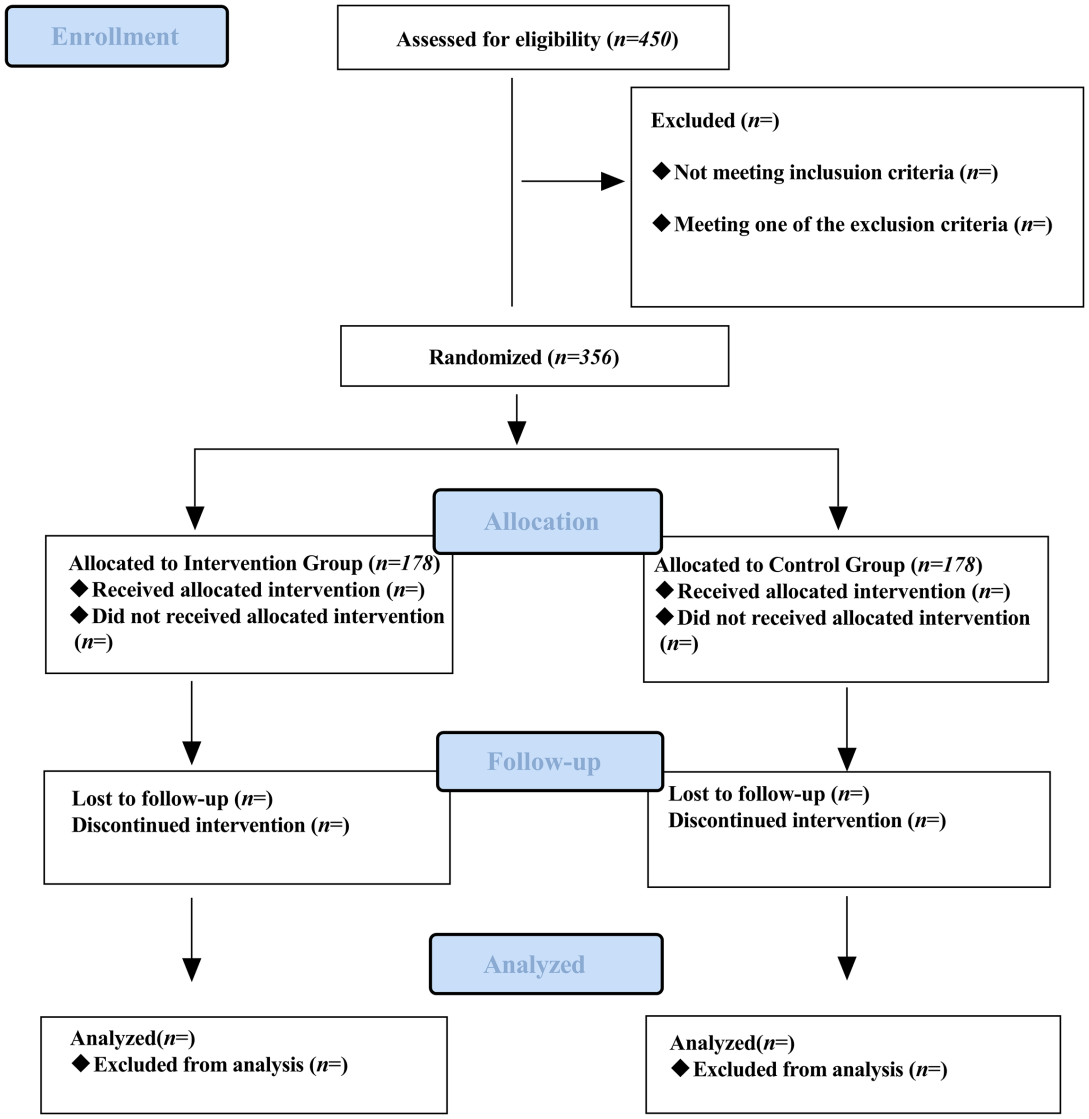
Flow chart of participant enrollment, intervention, assessments, and visits.

This single-center, prospective, double-blind, randomized, placebo-controlled trial will be conducted at the West Coast Hospital of the Affiliated Hospital of Qingdao University. Eligible patients will be randomly assigned to the esketamine or placebo group at a 1:1 ratio (Figure 1).

### 2.2. Ethics statement

The Clinical Trial and Biomedical Ethics Committee of the Affiliated Hospital of Qingdao University has approved this trial. During the clinical trial, any protocol modifications must be filed with and approved by the Ethics Committee. Before participation, members of the research team will explain the study verbally and in writing and obtain each participant’s written informed consent. An investigator will completely and comprehensively describe the study purpose and methods, the drug’s effects, reasonably expected benefits, possible side effects, and potential risks to the participant or their designated agent. This clinical trial follows the Declaration of Helsinki, Good Clinical Practice (GCP) for Drug Clinical Trials as issued by the State Drug Administration, and other relevant regulations. It is registered in the China Clinical Trial Registration Center (ChiCTR2300069044).

### 2.3. Eligibility criteria

#### 2.3.1. Inclusion criteria

(1) Age 18–75 years; (2) body mass index (BMI) 18.5–31.9 kg/m^2^; (3) American Society of Anesthesiologists (ASA) class I–III; (4) meets TKA treatment standards including: (1) obvious knee joint pain with painless walking distance <500 meters, (2) poor effects from conservative treatment, and (3) imaging findings show apparent knee joint damage; and 4–specific disease presence including rheumatoid arthritis, osteoarthritis, traumatic arthritis, or primary or secondary osteochondral necrosis.

#### 2.3.2. Exclusion criteria

(1) hemophilic arthritis or Charcot artropothy; (2) previous ipsilateral knee joint surgical history; (3) severe cardiocerebrovascular disease (e.g., hypertension grade III, serious valvular disease, chronic heart failure, severe arrhythmia, ischemic heart disease, stroke with apparent symptoms); (4) severe liver or kidney disease (e.g., Child–Pugh score III, creatinine clearance rate < 35 ml/min); (5) nervous or mental illness; (6) past or current intracranial hypertension; (7) allergy to local anesthetics; (8) infection at the puncture site; (9) declining to participate or unable to understand written study information; (10) glaucoma; (11) hyperthyroidism with no or ineffective treatment; (12) obstructive sleep apnea (STOP-BANG score ≥ 5).

### 2.4. Drop-out criteria

Withdrawal by the investigator will occur in the events of: (1) the investigator considers it necessary to stop the trial for medical ethics reasons; (2) the participant has a severe adverse event (SAE) and their continued participation is unsuitable; (3) the investigator judges that study withdrawal is in the participant’s best interest; (4) poor study adherence by the participant.

The participant will also have the right to withdraw their consent to participate in the trial at any time, as described in their informed consent. They will also be considered to have withdrawn if they no longer accept the intervention and/or assessments or are lost to follow-up (also referred to as ‘withdrawal’ or ‘drop-out’). Reasons for drop-out will be determined to the extent possible and recorded.

### 2.5. Recruitment

We plan to recruit participants for this single-center clinical trial from the Affiliated Hospital of Qingdao University through a combination of on-site and community-based methods. In clinical diagnosis and treatment, surgeons can invite the patients who they expect meet the trial conditions to participate, and anesthesiologists will use posters and leaflets in the joint surgery outpatient clinic to inform patients about the clinical trial. Qingdao University serves a large territory with a large, permanent population. The number of joint surgery clinics at the Affiliated Hospital of Qingdao University ranks among the top in the country. Therefore, a large number of potential participants are expected to meet the inclusion criteria.

### 2.6. Sample size calculation

The sample size was calculated based on the primary outcome. According to previous studies, the incidence of severe postoperative pain in patients undergoing TKA with multimodal analgesia is 25–40% (16, 17). Considering variance in multimodal analgesia and follow-up times, we reviewed the records of 20 patients who had undergone unilateral TKA in the past month. With multimodal analgesia, 48% of patients reported severe pain (NRS ≥ 7 points) within 12 postoperative hours. Therefore, we assumed that the incidence of rebound pain in patients undergoing TKA in the context of multimodal analgesia is 45%. We expect that the incidence of rebound pain in the esketamine group will be at least 35% lower than in the placebo group during the first 12 post-surgical hours. Using Power Analysis and Sample Size version 15.0 (Stata Corp. LP, College Station, TX, United States) and assuming a significance (*α*) level = 0.05 and power (1 − *β*) = 80% determined sample sizes for the esketamine group (*N*_1_) = 160 and placebo group (*N*_2_) = 160. Considering potential withdrawal (10%), a final 356 patients (178 in each group) will be included to achieve the appropriate power to test our hypothesis.

### 2.7. Randomization and allocation concealment

Participants will be assigned a study number (1–178) in order of their enrollment. We will use block randomization to assign participants to groups at a 1:1 ratio with a block length of 4. Statistical Package for Social Sciences (SPSS) version 25.0 will be used to generate and record a random number series from 1 to 6, and then arrange the blocks accordingly. Randomized allocation will be performed by the clinical trial center, which will not be involved in other aspects of the trial. The randomization protocol will be placed in a sealed, opaque envelope, and the quality controller (Fang Sheng) will open the envelope in order of grouping to determine each participant’s assignment.

### 2.8. Double blinding

This study will follow a double-blind design. According to the allocation protocol, participants will be randomized to receive a continuous infusion of esketamine or 0.9% normal saline during the operation. To ensure investigator blinding, the drug administrator (Guilin Liu) will prepare syringes (Weigao Group Medical Polymer Products Co., Ltd., Weihai, Shandong, China) in a separate room, labeling them A or B. One syringe will be filled with 50 mg of esketamine and 0.9% of normal saline (20 ml total) and the other will be filled with the same volume of 0.9% normal saline. The appearances of syringes A and B will be identical, other than label. The drug administrator will be unaware of the distribution plan and the intervention measures accepted by the participants. Because esketamine has sedative and hypnotic effects, to prevent unblinding the investigators and participants, all participants will be given preoperative 0.05 mg/kg intravenous midazolam. Throughout data collection, nurses, surgeons, outcome assessors, and statistical analysts will all be unaware of participant treatment group assignments. An independent data and safety supervision committee will monitor the trial, without disclosing participant grouping, until completion of statistical analyzes.

### 2.9. Self-reported pain scores

Rebound pain will be defined based on self-reported pain score. The participant’s pain score will be recorded in a pain diary prepared by the clinical trial center. An investigator (Qun Li) will train the participants to use the pain diary and patient-controlled intravenous analgesia (PCIA) device 1 day before their operation. The pain diary consists of three parts: first, basic participant information (e.g., name, age, sex, height, weight, complications); second, operation and anesthesia information (e.g., surgical procedure, anesthesia procedure, postoperative analgesia mode, prescription analgesics, related analgesia pump parameters); third, the NRS and recording times. The anesthesiologist will complete the first and second parts during the operation; the participant will complete the third part.

**Figure 2:**
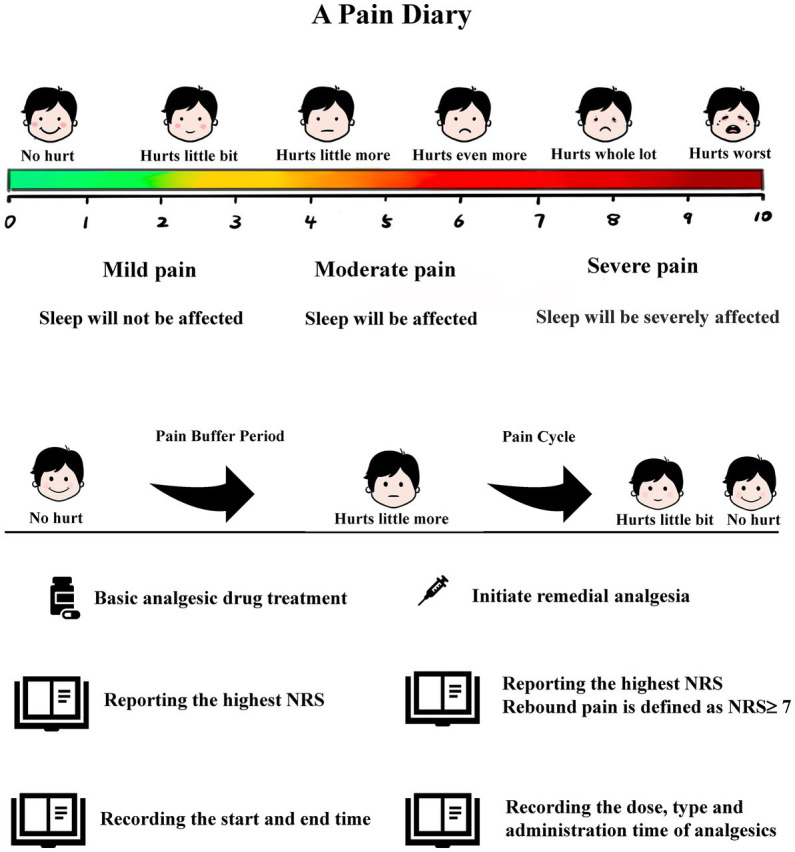
Participants report pain scores using a pain diary.

The pain diary stipulates that when the participant first experiences postoperative pain, they must accurately rate their pain severity and record the rating time using the pain face scale and ruler. The ward nursing staff will judge whether to start remedial analgesia according to the participant’s pain score. If the participant does not need remedial analgesia when the first pain occurs, they will also record their highest degree of pain during the first pain buffer period. The pain buffer period is defined as the time elapsed from tolerable pain (NRS < 4) to needing analgesic rescue intervention (NRS ≥ 4). If the participant has received remedial analgesia, they will record their highest degree of pain during the first pain cycle. The pain cycle is defined as the time elapsed from the pain requiring analgesic rescue intervention to when the pain is tolerable (NRS < 4). For each pain or buffer cycle, the participant will report their highest NRS and each cycle’s start and end time, until 24-h postoperative. The type, dose, and time of remedy analgesics given by the nursing staff will need to be accurately recorded in the pain diary (Figure 2).

### 2.10. Ultrasound-guided adductor tube block procedure

**Figure 3:**
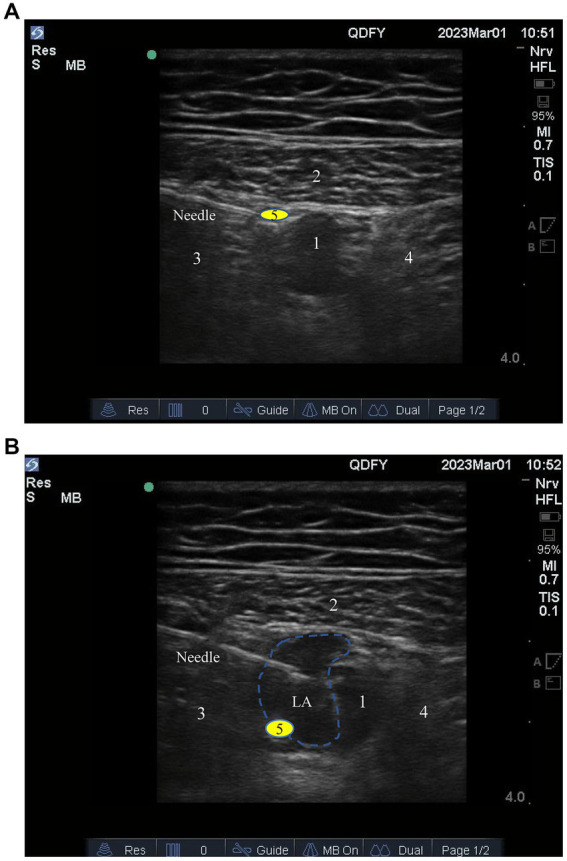
**(A)** The target for ultrasound-guided adductor canal block; **(B)** Diffusion of local anesthetic drugs within the adductor canal. 1, Femoral artery; 2, Sartorius muscle; 3, Medial vastus muscle; 4, Adductor magnus; 5, Saphenous nerve.

Before anesthesia, each participant will receive an ultrasound-guided adductor tube block in the nerve block room. Their peripheral venous access will be opened, and they will undergo three-lead electrocardiography (ECG), noninvasive blood pressure, and blood oxygen saturation monitoring. The participant will be placed supine, and the inner thigh of the affected side will be turned outward for full exposure. Polyvinylidene disinfectant (Nanjing Zhonghe Pharmaceutical Co., Ltd., Xuzhou, Jiangsu, China) will be used for skin disinfection. Sterile protective cover (Puen, Yangzhou Yutuo Medical Instrument Co., Ltd., Yangzhou, Jiangsu, China) will be used to cover high-frequency linear array probes (8–14 MHz). First, the anesthesiologist will place the probe at the anteromedial end of the middle thigh to find the short-axis section of the femoral artery. When the probe is positioned correctly, the skin, subcutaneous tissue, sartorius muscle, medial femoral muscle, and adductor magnus can be seen under ultrasound. At this position, the femoral artery, vein, and saphenous nerve will be located in the space surrounded by three muscles (Figure 3A). The anesthesiologist will use the in-plane technique to inject the needle from the outside to the inside toward the femoral artery. The needle tip target position is between the sartorius muscle, medial femoral muscle, and anterolateral side of the femoral artery. After the puncture needle breaks through the adductor femoris muscle membrane and its tip reaches the target position and no blood is drawn back, 1 ~ 2 ml of normal saline will be injected to confirm the diffusion position, after which 20 ml of 0.375% ropivacaine (AstraZeneca Pharmaceutical Co., Ltd., Wuxi, Jiangsu, China) will be injected. The sign of a successful block is that under ultrasound, the injected liquid can be seen surrounding the artery and spreading in the adductor tube like an ellipse (Figure 3B). Twenty 20 min after the block, the participant will be transferred to the operating room.

### 2.11. Spinal anesthesia procedure

After completing the adductor tube block, the participant will be placed in a lateral recumbent position (surgical side downward). The back of their spine will be close to the edge of the operating table and perpendicular to the ground. Their head will be bent forward and cushioned with a high pillow, their back curved, and their knees held with both hands. The anesthesiologist will locate the intersection between the line of the highest point of the posterior superior iliac spine and the spine: the fourth lumbar spine process (L_4 ~ 5_ space). The common puncture points are the L_3 ~ 4_ or L_2 ~ 3_ space. The anesthesiologist will choose the direct or lateral approach. The direct approach requires local infiltration anesthesia at the midpoint of the puncture space. The left thumb is fixed on the skin, and the right hand holds the No. 9 subarachnoid puncture needle (Henan Tuoren Medical Equipment Group Co., Ltd., Changheng, Henan, China). After passing through the skin, subcutaneous tissue, and supraspinous and interspinous ligaments, the needle is inserted with both hands. Resistance increases when the needle meets the ligamentum flavum. Cerebrospinal fluid outflow from the aspiration core confirms entry into the subarachnoid space. The puncture point for the side entry method should be 0.5 cm from the central line, and the puncture needle should be inclined 15° to the central line, with other procedures the same as the direct entry method. After confirming the free flow of cerebrospinal fluid through the needle, 10–15 mg of heavy gravity ropivacaine are administered. Post-injection, the participant will maintain the lateral position for 3–5 min, then turn and lie supine. The anesthesiologist will check for sensory changes and disappearance area, adjusting the participant’s body position as appropriate.

### 2.12. Interventions

Placebo group participants will receive an intravenous infusion of 0.9% normal saline (Weigao Group Co., Ltd., Weihai, Shandong, China). The infusion pump (Henan Tuoren Medical Equipment Group Co., Ltd., Changheng, Henan, China) mode is constant speed pumping, and the pumping time is from the end of the intraspinal anesthesia administration to the knee joint suture. The anesthesiologist can choose the appropriate medication protocol according to clinical practice, but other NMDA receptor antagonists, such as dextromethorphan and amantadine, will not be used.

Esketamine group participants will receive an intravenous infusion of esketamine (Jiangsu Heng Rui Medicine Co., Ltd., Jiangsu, China) following a standard dilution protocol [2 ml (50 mg) esketamine with 0.9% normal saline to 20 ml, 2.5 mg/ml]. The infusion rate of esketamine is 0.3 mg/kg/h, the mode of the infusion pump is constant speed pumping, and the infusion time is from the end of the spinal anesthesia administration to the knee joint suture. Drug managers need to monitor that the maximum dose of esketamine is not more than 0.5 mg/kg.

The esketamine dose selection was based on published clinical trials and drug manufacturer instructions. Koppert et al. (18) selected 13 healthy adult men to establish a pain model by inducing persistent pain and secondary mechanical hyperalgesia via transcutaneous electrical stimulation. In the trial group, 0.3 mg/kg/h esketamine was continuously infused for 30 min, and the observation index was the area of persistent pain, punctate hyperalgesia, and abnormal pain. The size of punctate hyperalgesia was significantly reduced and did not affect the hyperalgesia area. All participants felt comfortable after the procedure, quickly answered the investigators’ questions, and no anesthesia emergence was observed. For select major laparotomies (colorectal and liver surgeries), Bornemann-Cimenti et al. (14) formulated two drug delivery protocols: low-dose esketamine (no initial dose, followed by continuous infusion of 0.015 mg/kg/h for 48 h) and conventional low-dose esketamine (initial dose of 0.25 mg/kg, followed by continuous infusion of 0.125 mg/kg/h). The outcome indicators were opioid consumption, hyperalgesia, and postoperative delirium. Both protocols reduced postoperative opioid use and hyperalgesia, but the conventional low-dose group was more prone to delirium compared with the low-dose group. Trimmel et al. (19) showed that low-dose esketamine 0.125–0.25 mg/kg could produce analgesic effects with minimal incidence of adverse mental reactions. Nevertheless, adverse mental reactions can be as high as 12% at high doses. When the dose is increased to 0.5 ~ 1.0 mg/kg, separate anesthesia and adverse reactions increase accordingly. Increasing evidence suggests that esketamine should be used as an analgesic in adults to reduce postoperative opioid use. While esketamine doses were inconsistent in these studies, continuous perioperative infusion seems to be most effective for controlling postoperative pain.

TKA surgery in our center is expected to last 1–1.5 h. Due to the controlled use of psychotropic drugs, we plan to administer esketamine by continuous infusion at 0.3 mg/kg/h during surgery. This dose is lower than the specified dose in the manual and has analgesic and anti-hyperalgesia effects without causing adverse reactions like separation anesthesia. To ensure investigator and participant blinding and reduce mental symptoms, all participants will receive midazolam 0.05 mg/kg after spinal anesthesia. Before the operation, all participants will receive 0.5 mg of phenazopyridine hydrochloride (Chengdu List Pharmaceutical Co., Ltd., Chengdu, Sichuan, China) to antagonize the effect of esketamine on the M-cholinergic receptor.

### 2.13. Strategies to improve intervention adherence

At the recruitment and screening stage, the investigator will describe the study purpose, drug intervention, research protocol, trial process, drug delivery plan (including dose, delivery method, and time period), clinical assessments, sampling frequency, biological sample collection, potential risks and benefits of participation, and compensation, to ensure each participant is fully informed before they volunteer, and to enhance participant adherence. Before the participant begins the trial, the anesthesiologist, quality controller, and drug administrator will carefully check their participant number, randomization group, dosage, and pump speed. After the intervention, these investigators will carefully count the remaining research drugs, empty packages, and drug delivery devices.

### 2.14. Standard analgesia procedure

Each participant will be given 200 mg of celecoxib orally the day before their surgery. Twenty minutes before the end of the operation, the anesthesiologist will administer 4 mg of ondansetron (Qilu Pharmaceutical Co., Ltd., Jinan, Shandong, China). After the procedure, a PCIA device will be connected, loaded with 100 μg of sufentanil (Yichang Renfu Pharmaceutical Co., Ltd., Yichang, Hubei, China), 8 mg of ondansetron, and 5 mg of butorphanol tartrate (Jiangsu Heng Rui Medicine Co., Ltd., Jiangsu, China), diluted to 100 ml with normal saline. The background PCIA dose will be 1 ml/h, the single press dose will be 2 ml, the locking time 15 min, and the maximum infusion rate is 10 ml/h.

In the post-anesthesia care unit (PACU), when the participant’s NRS is ≥4 points (i.e., they require analgesics), the anesthesiologist will administer 50 μg of fentanyl (Jiangsu Nhwa Pharmaceutical Co., Ltd., Xuzhou, Jiangsu, China) intravenously as remedial the analgesia. The subsequent dose will be half of that preceding, at a between-rescue analgesia dose interval of ≥20 min. When the NRS of the participant is <4 points, the remedial analgesia is considered effective. In the surgical ward, the participant will receive flurbiprofen axetil 50 mg (Beijing Taide Pharmaceutical Co., Ltd., Beijing, China) twice daily and acetaminophen oxycodone (Fujian Minglong Pharmaceutical Co., Ltd., Longyan, Fujian, China) three times daily as the basic analgesic. The standard of care is to initiate the remedial analgesia procedure when the participant’s NRS is ≥4, at which time they are administered an intravenous injection of 50 mg of pethidine (Qinghai Pharmaceutical Factory Co., Ltd., Xining, Qinghai, China), which can be repeated every 4–6 h, for a maximum daily dose of 300 mg. In case of moderate to severe nausea or vomiting after the operation, 4 mg of intravenous ondansetron will be administered.

## 3. Outcomes

### 3.1. Primary outcome

The primary trial outcome is incidence of rebound pain within the first 12 postoperative hours in the esketamine and placebo groups. Herein, rebound pain is defined as the transition from well-controlled pain (NRS ≤ 3) to severe pain (NRS ≥ 7) in participants undergoing TKA under regional block anesthesia.

### 3.2. Secondary outcomes

1. Incidence of rebound pain within the first 24 postoperative hours.
2. Time to entering the first pain cycle during the first 24 postoperative hours (i.e., time from PACU transfer to initial analgesic intervention).
3. Time to first rebound pain during the first 24 postoperative hours (i.e., time from PACU transfer to first NRS ≥ 7).
4. The modified rebound pain score (MRPS). MRPS = HNRS−LoNRS (PACU) (2), where HNRS (24) is the highest NRS pain score within 24 h after regional nerve block, and LoNRS (PACU) is the lowest NRS pain score while in the PACU.
5. NRS scores at rest (i.e., laying quietly) and exercise (i.e., raising the affected limb ≥5 times to ≥30 cm) at: 1 day before their operation, PACU transfer, the morning of the first postoperative day; during physical therapy on the first postoperative day; the evening of the first postoperative day; the morning of the second postoperative day; and the evening of the second postoperative day. Morning is defined as 8–10 a.m. and evening as 8–10 p.m.
6. The cumulative opioids upon transfer to the PACU and at 12, 24, and 48 postoperative hours. Cumulative opioids includes administration of basic and remedial analgesics, which will be converted to ‘morphine-equivalent dose’ for analyzes.
7. One day before their operation and first postoperative Timed Up and Go test (i.e., time required to stand up from a standard-height armchair, walk forward three meters, walk back to the chair, and sit down).
8. Range of motion [i.e., degree of the affected knee joint from neutral (0°) to maximum flexion] of the active and passive knee joints at 1 day before their operation and on the first postoperative day.
9. Time to discharge (i.e., when the participant can walk from bed to bathroom independently and along the corridor without the help of a walker).
10. Length of stay (i.e., number of days from admission to discharge).
11. Quality of Recover (QOR)-15 scores at 1 day before their operation and the first postoperative day.
12. Blood glucose and cortisol concentration at admission and the first postoperative day.
13. Patient satisfaction at discharge, where 1 point is ‘unsatisfactory’, 2 points is ‘relatively satisfactory’, and 3 points is ‘satisfactory’.

### 3.3. Safety outcomes

(1) nausea and vomiting; (2) respiratory depression (i.e., blood oxygen saturation < 93%); (3) hypertension (i.e., blood pressure > 30% of baseline value); (4) hypotension (i.e., blood pressure < 30% of baseline value); (5) tachycardia (i.e., heart rate > 100 beats/min); (6) bradycardia (i.e., heart rate < 50 beats/min); (7) nervous system complications (restlessness, delirium, nightmare, restlessness); (8)increased secretions; (9) nerve injury caused by regional block or local anesthetic poisoning; (10) wound infection, deep vein thrombosis, or pulmonary embolism.

### 3.4. Trial safety

To ensure participant safety throughout the clinical trial, all inclusion and exclusion criteria will be strictly adhered to. The day before their operation, an investigator (Qun Li) will describe the study purpose and protocol, drug effects, potential benefits, possible toxic and side effects, and potential risks to the participant or their designated agent. Before participation, the research team will obtain the written informed consent of each participant to protect their legitimate rights and interests. Before starting this trial, the investigator will review the emergency plan for improvements and will communicate with multiple departments regarding adverse events (AE) that may occur in this clinical study to arrange protocols for their assistance in case of emergency. The nerve block technique is conducted under ultrasound guidance and is administered by an experienced expert. During and after the nerve block operation, participants will undergo ECG, blood oxygen, and blood pressure monitoring. The dose and concentration of esketamine administered to each study participant will be based on evidence-based standard of clinical care and drug manufacturer recommendations. A multifunction monitor will be used to monitor participants during surgery. All anesthesiologists participating in this trial have rich clinical experience, are skilled at handling critical events, and have received professional GCP training. The quality control nurse will check whether first aid drugs and materials are complete and within their periods of validity before the trial. Our medical institution has developed standard treatment and rescue procedures for adverse reactions caused by medications, including nausea and vomiting, psychiatric symptoms, and anaphylactic shock. In the PACU and surgical wards, participants will undergo continuous ECG, blood oxygenation, and blood pressure monitoring for at least 24 h. The Medical Ethics Committee and the Clinical Trial Safety Supervision Committee of Qingdao University will supervise the trial.

### 3.5. Adverse event reporting

AE in this trial include any adverse symptoms, abnormal physical signs, and abnormal experimental results after the intervention, regardless of whether there is a causal relation with the intervention. Any AE, including those described by the participant or obtained through investigator inquiry or physical examination, laboratory examination, or other examination methods, will be recorded, actively managed, and closely tracked until the condition is relieved or stable. The investigator will track all AE and record them in the standard paper case report form (CRF), including their detailed content, location, time, severity, treatment measures, and treatment results.

SAE refers to death, life-threatening, severe disability or loss of function, need for hospitalization or prolonged hospitalization, and other critical medical events after the participants receive the intervention. Should a SAE occur, all rescue efforts will be made and necessary measures taken to actively avoid permanent damage. The investigator will notify the person in charge, the clinical trial organization, and the ethics committee by telephone or fax within 24 h and record the SAE details in the CRF.

## 4. Data collection

An investigator (Qun Li, Lili Jiang, or Guilin Liu) will conduct a preoperative visit with eligible participants the day before their surgery. Baseline data, recorded from participant self-report and their electronic medical records, will include age, sex, height, weight, ASA grade, complications, duration of analgesic use, admission time, and preoperative blood glucose and cortisol concentration. The NRS will be used to assess baseline pain. The Timed Up and Go test will be used to evaluate baseline knee function and active and passive knee flexion. Preoperative baseline recovery quality score will be evaluated using the QOR-15. The anesthesiologist will collect and record intraoperative data through the anesthesia management system, including dosages of sedatives and analgesics, steroid use (yes/no), blood loss, infusion volume, tourniquet use time, operation time, and any AE. An investigator (Xinyue Wang, Wenhan Zhang, Tangwen Xu, or Luyong Wang) will collect postoperative data, including pain-related indicators, opioid consumption, knee joint function recovery indicators, and any AE. Postoperative data will be collected from the doctor and nurse management stations of the hospital information system, standardized CRF, and PCIA information storage cards (Table 1). Outcome assessors will have been trained in the standard operating procedures of clinical drug trials. The clinical data monitoring committee (DMC) will supervise all data collection to ensure reporting accuracy, integrity, readability, and timeliness. All source files will be rigorously cleaned to ensure accurate data use.

**Table 1:** Study protocol.

Outcome measure	Visit 1	Visit 2	Visit 3	Visit 4	Visit 5	Visit 6	Visit 7
Time	1 day pre- surgery	During operation	PACU	Within 12 h post-surgery	POD 1	POD 2	Hospital discharge
Eligibility screening	X	–	–	–	–	–	–
Informed consent	X	–	–	–	–	–	–
Demographic characteristics	X	–	–	–	–	–	–
Preoperative education	X	–	–	–	–	–	–
Baseline measures	X	X	–	–	–	–	–
Randomization	X	–	–	–	–	–	–
Intervention	–	X	–	–	–	–	–
Incidence of rebound pain	–	–	–	X	X	–	–
First time to begin pain cycle	–	–	–	X	X	–	–
First time rebound pain occurred	–	–	–	X	X	–	–
NRS scores	X		X	X	X	X	–
Cumulative opioid consumption	–	X	X	X	X	X	–
Timed Up and Go test	X	–	–	–	X	–	–
Range of motion of knee joints	X	–	–	–	X	–	–
Time to reach discharge standard	–	–	–	–	–	–	X
Length of stay	–	–	–	–	–	–	X
Blood glucose and cortisol concentration	X	–	–	–	X	–	–
Patient satisfaction at discharge	–	–	–	–	–	–	X
Safety outcomes	Throughout the study period, until patient discharge						

NRS, numeric rating scales; POD, postoperative day.

## 5. Data management

An electronic data capture (EDC) system will be used for data management. The data management process will follow GCP for clinical drug trials and relevant national laws and regulations, guidelines, the data management plan (DMP), and appropriate data management standard operating procedures. The clinical research protocol stipulates that the data manager (Xue Sun) will write the DMP to standardize the data management process. The DMP is a dynamic file that can be modified and updated according to the actual situation during the study. The electronic CRF (eCRF) will be designed by the data manager according to the clinical study protocol and reviewed by the clinical project leader (Yang Zhao) and the statistician. These professionals will be responsible for setting up the EDC database and configuring logical verification rules. After the configuration is completed, study professionals will conduct online tests, including data entry, data export, logical check tests, eCRF interface tests, and system function tests, to confirm the accuracy and rationality of the database structure and input page. The data manager will write a data verification plan according to the clinical study protocol and eCRF.

Before data entry, the data manager will train relevant personnel on using the uniform EDC. The clinical research coordinator (CRC) authorized by the project leader will log into the EDC through an independent account and enter the participant’s data according to study protocol. All data will be verified for accuracy. Timely data entry and backup will be maintained throughout the clinical research data management process. The database will be deployed on the Ali Cloud server, with automatic daily backups at a fixed time. After data cleaning, the data manager will write a data review report and then organize all investigators, clinical project leaders, and statisticians for a data review meeting and confirm dataset divisions. Any data problems will be efficiently corrected by the responsible person. After verifying that all lock list items are complete, all investigators, clinical principals, data managers, and statisticians will sign the lock approval document, and the database will be locked. All necessary records will be kept in a secure, confidential location, readily available for access and inspection by relevant authorities.

## 6. Data monitoring

The DMC will include the head of the research institution, clinical inspector, and statistician. The DMC will conduct timely data verification according to the established verification plan. Data verification problems will be posed online and addressed by the investigator or their authorized CRC. The DMC will audit the implementation of the trial-related documents, trial data, and trial protocol, as planned, and send audit results to the Clinical Trial Center of Qingdao University and the Medical Ethics Committee. The audit may occur at any time during or after the trial. The investigator will allow the clinical inspector access to all relevant documents.

## 7. Statistical methods

We plan to include data from all the participants assigned to randomized grouping, regardless of intervention, in a intention-to-treat analysis. SPSS version 25.0 will be used to analyze data. The Shapiro–Wilk test will be used to assess normality of continuous variables. Normal data will be described as means ± standard deviations, and otherwise as medians and interquartile ranges. Demographic and intraoperative data will be compared by independent samples *t*-tests, Mann–Whitney U test, chi-square test, Yates’ chi-square test, or Fisher’s exact probability test, as appropriate. The primary study outcome (rebound pain) is dichotomous, and the relative risk (RR) and 95% confidence interval (CI) will be calculated using the modified Poisson regression model for the esketamine and placebo groups. We plan to evaluate three modified Poisson regression models: model 1 will be unadjusted; model 2 will be adjusted for gender, age, BMI, ASA grade, complications, long-term analgesics use, and baseline pain score; model 3 will be adjusted for tourniquet use time, operation time, steroid use (yes/no) and analgesic and sedative doses during the operation. We plan to conduct a sensitivity analysis on the primary outcome. The effects of the intention-to-treat set and the per-protocol set on the consistency of the primary outcome will be compared. The per-protocol set will be whether the participant has completed all main baseline variables, has not violated the inclusion or exclusion criteria, has completed all trials and assessments, and was in good adherence. Second, we plan to compare the impacts of different statistical methods (i.e., stratified chi-square test and modified Poisson regression model) on the consistency of the primary outcome. Stratified chi-square analysis adjusted for major potential confounders, i.e., age, sex, use of steroids (yes/no), preoperative pain score, and long-term use of analgesics (yes/no) before surgery. Adjusted OR values will be calculated using the Cochran–Mantel–Haenszel test (CMH).

Secondary outcomes will be exploratory, without adjustment for multiple comparisons. The first time to enter the pain cycle within the first 24 postoperative hours and the first time to rebound pain within the first 24 postoperative hours will constitute the survival data. Kaplan–Meier survival curves and log-rank tests will be used to analyze survival data, and univariate COX regression will be used to calculate HR and 95% CI. NRS and opioid use at different postoperative intervals will constitute repeated-measurement data. Generalized estimation equation (GEE) will be used, with patient identification number as the main variable, time as the main internal variable, and NRS and opioid consumption as the dependent variables. Independent samples *t*-tests or Mann–Whitney U tests will be used to compare independent data between the groups. The Hodges–Lehmann method will be used to evaluate the pseudo-median differences and 95% CI. Categorical data will be expressed as the number of cases (percentage) and analyzed using the chi-square test, Yate’s chi-square test, or Fisher’s exact probability test. Safety outcomes will be analyzed using the safety analysis dataset. All participants who undergo randomization and for whom post-trial safety evaluation data are available will be included in the safety analysis dataset. All statistical tests will be two-sided and *p* < 0.05 will be considered statistically significant.

### 7.1. Missing data handling

The principle of intention-to-treat analysis will be used herein. Participant data may be missing due to study withdrawal, loss to follow-up, excessive side effects, or other reasons. Missing data will be processed by multiple imputations, with the imputation times set to 5 and 50 maximum iterations. A predictive mean matching model will be used for imputing continuous variables, and a logistic regression model for imputing categorical variables. After imputation, the final data will be analyzed.

## 8. Discussion

For this single-center, prospective, double-blind, randomized, placebo-controlled trial, we expect that rebound pain occurrence within 12 h after TKA will be significantly reduced in the esketamine group compared with the placebo group. The analgesic effect of ketamine may be through inhibiting NMDA receptors in nociceptive neurons and activating the monoaminergic descending inhibitory pain pathway (20). NMDA receptors play an active role in the nociceptive process of spinal dorsal horn ganglia (21, 22). Low-dose ketamine can alleviate pain by reducing NMDA receptor-mediated secondary hyperalgesia and central sensitization, and can reduce opioid-induced hyperalgesia by interacting with opioid receptors (20). Central sensitization is a phenomenon in which the duration and amplitude of neuronal responses in the spinal dorsal horn increase during repetitive, constant-intensity C-fiber stimulation. Animal studies have shown that blocking NMDA receptors prevents pain sensitivity and opioid tolerance. In addition, inhibition of large conductance Ca^2+^-activated K^+^ channels in microglia may contribute to the analgesic effect of ketamine (23). A low dose of esketamine effectively reduces opioid consumption and hyperalgesia after abdominal surgery, and may reduce the risk of postoperative delirium (14). PCIA with combined esketamine and oxycodone can reduce cumulative oxycodone consumption during the first 24 h after lumbar fusion surgery without adverse reactions (24). Esketamine has also shown significant effects compared with placebo for preventing acute pain syndrome after thoracotomy (25). Esketamine can effectively control postoperative pain in women undergoing cesarean section, reduce opioid dosages, and improve rehabilitation quality (26). However, postoperative pain improvements are dissimilar between low and high esketamine doses. Given the potential AE associated with esketamine, a low dose may be more appropriate for postoperative pain control (14, 26).

Clinically, rebound pain and breakout pain are very similar, generally manifesting as sudden and severe pain after surgery. Breakout pain was originally only used to describe cancer pain, but pain with similar characteristics is now recognized as widespread among non-cancer populations. Breakout pain has been observed in neoplastic and non-neoplastic patients without background pain. Though many definitions of breakout pain have been described in different literatures, the most internationally accepted definition is that of the European Association for Palliative Care, “A transient increase in pain that occurs spontaneously or is associated with a specific predictable or unpredictable trigger despite a background pain that is relatively stable and adequately controlled.” However, there is currently no valid method for diagnosing post-surgical breakout pain. The factors influencing breakout pain are complex, including anesthesia methods, surgical methods, postoperative analgesia regimens, and patient factors (27, 28). ‘Rebound pain’ is used to describe the transient, acute postoperative pain that occurs after the resolution of the sensory block from regional anesthesia and after peripheral nerve blocks and intraspinal anesthesia. The essential rebound pain mechanisms may be sudden exposure to nociceptive pain from insufficient prior analgesia in multimodal analgesia and hyperalgesia induced by regional anesthesia and surgical trauma. As a non-competitive NMDA antagonist, esketamine reduces postoperative opioid consumption and decreases the hyperalgesia area. Therefore, using rebound pain as an indicator herein is most appropriate in terms of its definition, intervention, and participant population.

This study will compare the effects of esketamine on the rebound pain occurrence after resolution of regional block in TKA patients. The trial has five known potential limitations. First, it will only use a single esketamine dose for observation and lack of parallel control between different doses; the optimal dose has yet to be determined, thus the trial may yield negative results. Second, the NRS will be the primary tool for assessing rebound pain. Participant report may be based on pain recall, which is subjective and possibly biased. Third, due to drug safety issues, esketamine will be administered only during the procedure, not added to the intravenous analgesia pump or continuously infused after surgery. Fourth, the sample size will be limited and will be conducted at a single center. Multicenter replication will be needed to standardize practices and increase reliability and generalizability of the results. Finally, that all procedures will be performed by the same team of surgeons, using the same process, is both a strength and a weakness. It will be conducive to consistency and internal validity but may limit reproducibility.

## Ethics statement

The studies involving human participants were reviewed and approved by the Clinical Trial and Biomedical Ethics Committee of the Affiliated Hospital of Qingdao University. The patients/participants provided their written informed consent to participate in this study. Written informed consent was obtained from the individual(s) for the publication of any potentially identifiable images or data included in this article.

## Author contributions

YoZ, QL, and YaZ study design and planning. YoZ, QL, GL, FS, XZ, LJ, JH, BZ, and ZZ study conduct. QL, GL, FS, XZ, LJ, JH, SL, and ZZ data analysis. YoZ and QL writing paper: YoZ, QL, CW, XJ, and YaZ revising paper. All authors contributed to the article and approved the submitted version.

## Funding

This trial will be supported by the Qingdao 2022 Annual Medical and Health Research Guidance Project Fund (2022-WJDZ186) and 2021 Shandong Medical Association Clinical Research Fund–Qilu Special Project (YXH2022ZX02095). The funders will have no role in the design of the trial, the collection of the data, the statistical analysis, or the writing of the clinical trial.

## Conflict of interest

The authors declare that the research was conducted in the absence of any commercial or financial relationships that could be construed as a potential conflict of interest.

## Publisher’s note

All claims expressed in this article are solely those of the authors and do not necessarily represent those of their affiliated organizations, or those of the publisher, the editors and the reviewers. Any product that may be evaluated in this article, or claim that may be made by its manufacturer, is not guaranteed or endorsed by the publisher.

## References

[1] Hamilton DL. Rebound pain: distinct pain phenomenon or nonentity? Br J Anaesth. (2021) 126:761–3. doi: 10.1016/j.bja.2020.12.034, PMID: 33551124

[2] Barry GS Bailey JG Sardinha J Brousseau P Uppal V. Factors associated with rebound pain after peripheral nerve block for ambulatory surgery. Br J Anaesth. (2021) 126:862–71. doi: 10.1016/j.bja.2020.10.035, PMID: 33390261

[3] Kolarczyk LM Williams BA. Transient heat hyperalgesia during resolution of ropivacaine sciatic nerve block in the rat. Reg Anesth Pain Med. (2011) 36:220–4. doi: 10.1097/AAP.0b013e3182176f5a, PMID: 21451438 PMC3085276

[4] Muñoz-Leyva F Cubillos J Chin KJ. Managing rebound pain after regional anesthesia. Korean J Anesthesiol. (2020) 73:372–83. doi: 10.4097/kja.20436, PMID: 32773724 PMC7533186

[5] Li JW Ma YS Xiao LK. Postoperative pain Management in Total Knee Arthroplasty. Orthop Surg. (2019) 11:755–61. doi: 10.1111/os.12535, PMID: 31663286 PMC6819170

[6] Nobre LV Cunha GP Sousa P Takeda A Cunha Ferraro LH. Peripheral nerve block and rebound pain: literature review. Braz J Anesthesiol. (2019) 69:587–93. doi: 10.1016/j.bjan.2019.05.001, PMID: 31690509 PMC9391878

[7] Woo JH Lee HJ Oh HW Lee JW Baik HJ Kim YJ. Perineural dexamethasone reduces rebound pain after ropivacaine single injection interscalene block for arthroscopic shoulder surgery: a randomized controlled trial. Reg Anesth Pain Med. (2021) 46:965–70. doi: 10.1136/rapm-2021-102795, PMID: 34535548

[8] Zhou Q Yu L Yin C Zhang Q Tai Y Zhu L Jnr . Effect of Transauricular Vagus nerve stimulation on rebound pain after Ropivacaine single injection femoral nerve block for anterior cruciate ligament reconstruction: a randomized controlled trial. J Pain Res. (2022) 15:1949–58. doi: 10.2147/JPR.S370589, PMID: 35860416 PMC9292065

[9] Lee JJ Kim DY Hwang JT Song DK Lee HN Jang JS . Dexmedetomidine combined with suprascapular nerve block and axillary nerve block has a synergistic effect on relieving postoperative pain after arthroscopic rotator cuff repair. Knee Surg Sports Traumatol Arthrosc. (2021) 29:4022–31. doi: 10.1007/s00167-020-06288-8, PMID: 32975624 PMC7517062

[10] Hwang JT Jang JS Lee JJ Song DK Lee HN Kim DY . Dexmedetomidine combined with interscalene brachial plexus block has a synergistic effect on relieving postoperative pain after arthroscopic rotator cuff repair. Knee Surg Sports Traumatol Arthrosc. (2020) 28:2343–53. doi: 10.1007/s00167-019-05799-3 31773201

[11] Lee JJ Kim DY Hwang JT Lee SS Hwang SM Kim GH . Effect of ultrasonographically guided axillary nerve block combined with suprascapular nerve block in arthroscopic rotator cuff repair: a randomized controlled trial. Arthroscopy. (2014) 30:906–14. doi: 10.1016/j.arthro.2014.03.014, PMID: 24880194

[12] Williams BA Bottegal MT Kentor ML Irrgang JJ Williams JP. Rebound pain scores as a function of femoral nerve block duration after anterior cruciate ligament reconstruction: retrospective analysis of a prospective, randomized clinical trial. Reg Anesth Pain Med. (2007) 32:186–92. doi: 10.1097/00115550-200705000-00003, PMID: 17543812 PMC1940333

[13] Ding DY Manoli A Galos DK Jain S Tejwani NC. Continuous popliteal sciatic nerve block versus single injection nerve block for ankle fracture surgery: a prospective randomized comparative trial. J Orthop Trauma. (2015) 29:393–8. doi: 10.1097/BOT.0000000000000374, PMID: 26165259

[14] Bornemann-Cimenti H Wejbora M Michaeli K Edler A Sandner-Kiesling A. The effects of minimal-dose versus low-dose S-ketamine on opioid consumption, hyperalgesia, and postoperative delirium: a triple-blinded, randomized, active- and placebo-controlled clinical trial. Minerva Anestesiol. (2016) 82:1069–76. PMID: 27327855

[15] Touil N Pavlopoulou A Barbier O Libouton X Lavand'homme P. Evaluation of intraoperative ketamine on the prevention of severe rebound pain upon cessation of peripheral nerve block: a prospective randomised, double-blind, placebo-controlled study. Br J Anaesth. (2022) 128:734–41. doi: 10.1016/j.bja.2021.11.043, PMID: 35219449

[16] Andersen L Gaarn-Larsen L Kristensen BB Husted H Otte KS Kehlet H. Subacute pain and function after fast-track hip and knee arthroplasty. Anaesthesia. (2009) 64:508–13. doi: 10.1111/j.1365-2044.2008.05831.x, PMID: 19413820

[17] Grevstad U Mathiesen O Lind T Dahl JB. Effect of adductor canal block on pain in patients with severe pain after total knee arthroplasty: a randomized study with individual patient analysis. Br J Anaesth. (2014) 112:912–9. doi: 10.1093/bja/aet441, PMID: 24401802

[18] Koppert W Sittl R Scheuber K Alsheimer M Schmelz M Schüttler J. Differential modulation of remifentanil-induced analgesia and postinfusion hyperalgesia by S-ketamine and clonidine in humans. Anesthesiology. (2003) 99:152–9. doi: 10.1097/00000542-200307000-00025, PMID: 12826855

[19] Trimmel H Helbok R Staudinger T Jaksch W Messerer B Schöchl H . S(+)-ketamine: current trends in emergency and intensive care medicine. Wien Klin Wochenschr. (2018) 130:356–66. doi: 10.1007/s00508-017-1299-3, PMID: 29322377 PMC6061669

[20] Hirota K Lambert DG. Ketamine: new uses for an old drug? Br J Anaesth. (2011) 107:123–6. doi: 10.1093/bja/aer221, PMID: 21757548

[21] Ruscheweyh R Wilder-Smith O Drdla R Liu XG Sandkühler J. Long-term potentiation in spinal nociceptive pathways as a novel target for pain therapy. Mol Pain. (2011) 7:1744-8069-7-20. doi: 10.1186/1744-8069-7-20 PMC3078873 21443797

[22] Sandkühler J Gruber-Schoffnegger D. Hyperalgesia by synaptic long-term potentiation (LTP): an update. Curr Opin Pharmacol. (2012) 12:18–27. doi: 10.1016/j.coph.2011.10.018, PMID: 22078436 PMC3315008

[23] Brinck EC Tiippana E Heesen M Bell RF Straube S Moore RA . Perioperative intravenous ketamine for acute postoperative pain in adults. Cochrane Database Syst Rev. (2018) 12:Cd012033. doi: 10.1002/14651858.CD012033.pub4, PMID: 30570761 PMC6360925

[24] Brinck ECV Virtanen T Mäkelä S Soini V Hynninen VV Mulo J . S-ketamine in patient-controlled analgesia reduces opioid consumption in a dose-dependent manner after major lumbar fusion surgery: a randomized, double-blind, placebo-controlled clinical trial. PLoS One. (2021) 16:e0252626. doi: 10.1371/journal.pone.0252626, PMID: 34097713 PMC8183989

[25] Mendola C Cammarota G Netto R Cecci G Pisterna A Ferrante D . S(+)-ketamine for control of perioperative pain and prevention of post thoracotomy pain syndrome: a randomized, double-blind study. Minerva Anestesiol. (2012) 78:757–66. PMID: 22441361

[26] Wang Y Zhang Q Dai X Xiao G Luo H. Effect of low-dose esketamine on pain control and postpartum depression after cesarean section: a retrospective cohort study. Ann Palliat Med. (2022) 11:45–57. doi: 10.21037/apm-21-3343, PMID: 35144397

[27] Esparza-Miñana JM. Diagnosis and medical treatment of breakthrough pain. Med Clin (Barc). (2018) 150:114–8. doi: 10.1016/j.medcli.2017.10.001, PMID: 29157656

[28] Liossi C Greenfield K Schoth DE Mott C Jassal S Fraser LK . A systematic review of measures of breakthrough pain and their psychometric properties. J Pain Symptom Manag. (2021) 62:1041–64. doi: 10.1016/j.jpainsymman.2021.04.018, PMID: 33933619

